# Levosimendan versus dobutamine in Tako-tsubo cardiomyopathy

**DOI:** 10.1186/cc12165

**Published:** 2013-03-19

**Authors:** FR Righetti, MP Parolini, GC Castellano

**Affiliations:** 1 St. Boniface Hospital Verona, Italy

## Introduction

Tako-tsubo cardiomyopathy is a clinical entity characterized by a dysfunction of the left ventricle, usually transient, which manifests itself with symptoms that can mimic an acute coronary syndrome. It is characterized by transient ballooning modification of the left ventricular apex, probably due to neurogenic stimuli resulting in low cardiac output syndrome. The aim of this prospective randomized study was to evaluate, by serial transesophageal echocardiography (TEE), what is the best drug treatment between levosimendan and dobutamine to restore a satisfactory cardiac function in the case of low cardiac output.

## Methods

Twelve adult patients, aged 18 years, were admitted to the ICU with Tako-tsubo cardiomyopathy at entrance. The patients were divided randomly into two groups: levosimendan (six patients) treated with levosimendan and standard treatment, and the control group (six patients) with dobutamine and standard treatment. In all patients, serial TEE was performed studying the systolic function, by ejection fraction of the left ventricle with Simpson's method. The TEEs were performed at the entrance of the patient, after 12 and 24 hours of treatment. The results were expressed as mean with standard deviation. For the comparison between the two groups we used the Fisher test, considered significant with *P <*0.05.

## Results

Patients in the two groups were statistically comparable with respect to sex (*P *= 0.31) and age (*P *= 0.53). The causes of the syndrome of Tako-tsubo were: subarachnoid hemorrhage (six patients) after coronary artery bypass graft (four patients), and polytrauma (two patients). All patients had low cardiac output. In the levosimendan group the ejection fraction at entrance was 25 ± 6%, after 12 hours 36 ± 10%, and 47 ± 5% after 24 hours. In the control group the ejection fraction at entrance was 24 ± 7%, after 12 hours 28 ± 6% and after 24 hours 33 ± 4%. Comparing the two groups we reached statistical significance, *P *= 0.026.

## Conclusion

Comparing the two groups, we noticed that both started from a low cardiac output. However, in the group who used the drug therapy based on levosimendan we saw a return of systolic function of the left ventricle to near-normal levels within 24 hours, while in the control group there remains a dysfunction in systolic function. We have shown the drug therapy based on levosimendan contributes to improving the systolic function of the left ventricle compared with treatment with dobutamine despite the initial cardiac stunning.
